# Vitamin D receptor ApaI polymorphism associated with progression of liver disease in Vietnamese patients chronically infected with hepatitis B virus

**DOI:** 10.1186/s12881-019-0903-y

**Published:** 2019-12-21

**Authors:** Nghiem Xuan Hoan, Nguyen Khuyen, Dao Phuong Giang, Mai Thanh Binh, Nguyen Linh Toan, Do Tuan Anh, Ngo Tat Trung, Mai Hong Bang, Christian G. Meyer, Thirumalaisamy P. Velavan, Le Huu Song

**Affiliations:** 1 Institute of Clinical Infectious Diseases, 108 Institute of Clinical Medical and Pharmaceutical Sciences, 108 Military Central Hospital, Tran Hung Dao Street N1, Hai Ba Trung District, Hanoi, Vietnam; 2 Faculty of Tropical and Infectious Diseases, 108 Institute of Clinical Medical and Pharmaceutical Sciences, Hanoi, Vietnam; 3 Institute of Tropical Medicine, University of Tübingen, Tübingen, Germany; 4 Vietnamese-German Center for Medical Research (VG-CARE), Hanoi, Vietnam; 5 Department of Infectious Diseases, Duc Giang Hospital, Hanoi, Vietnam; 6 Department of Molecular Biology, 108 Institute of Clinical Medical and Pharmaceutical Sciences, Hanoi, Vietnam; 7 Department of Gastroenterology, 108 Institute of Clinical Medical and Pharmaceutical Sciences, Hanoi, Vietnam; 8 Department of Pathophysiology, Vietnam Military Medical University, Hanoi, Vietnam; 9 Department of Infectious Diseases, 103 Military Hospital, Hanoi, Vietnam; 10 Duy Tan University, Da Nang, Vietnam

**Keywords:** HBV, Hepatitis B, VDR, Polymorphism, Liver diseases

## Abstract

**Background:**

Vitamin D derivatives and their receptor (VDR) are potent modulators of immune responses in various diseases including malignancies as well as in metabolic and infectious disorders. The impact of vitamin D receptor polymorphisms on clinical outcomes of hepatitis B virus (HBV) infection is not well understood. This study aims to investigate the potential role of VDR polymorphisms (TaqI, FokI, ApaI, and BsmI) in Vietnamese HBV infected patients and to correlate these polymorphisms with the progression of HBV-related liver disease.

**Methods:**

Four hundred forty-three HBV infected patients of the three clinically well-defined subgroups chronic hepatitis B (CHB, *n* = 183), liver cirrhosis (LC, *n* = 89) and hepatocellular carcinoma (HCC, *n* = 171) and 238 healthy individuals (HC) were enrolled*. VDR* polymorphisms were genotyped by DNA sequencing and in-house validated ARMS assays. Logistic regression models were applied in order to determine the association of *VDR* polymorphisms with manifest HBV infection as well as with progression of related liver diseases mulin different genetic models.

**Results:**

The *VDR* ApaI CA genotype was less frequent in HCC than in CHB patients in different genetic models (codominant model, OR = 0.5, 95%CI = 0.3–0.84, *P* = 0.004; dominant model, OR = 0.46, 95%CI = 0.27–0.76, *P* = 0.0023). In the recessive model, the genotype *ApaI* AA was found more frequently among HCC compared to CHB patients (OR = 2.56, 95%CI = 1.01–6.48, *P* = 0.04). Similarly, the *ApaI* CA genotype was less frequent in HCC than in non-HCC group codominant model, OR = 0.6, 95%CI = 0.4–0.98, dominant model, *P* = 0.04 and OR = 0.6, 95%CI = 0.38–0.90, *P* = 0.017). The ApaI genotypes CA and AA was significantly associated with higher levels of liver enzymes, bilirubin, and HBV DNA (*P* < 0.05). No association between TaqI, FokI and BsmI polymorphisms and any clinical outcome as well as liver disease progression was found.

**Conclusions:**

Among the four investigated VDR polymorphisms, ApaI is associated with clinical outcome and liver disease progression in Vietnamese HBV infected patients.

## Background

Hepatitis B virus (HBV) infection remains a major health problem even with a high coverage of effective vaccination meanwhile achieved. The infection can be life-threatening due to its frequent severe complications, consisting of fulminant acute hepatitis, acute or chronic hepatitis (CHB), liver cirrhosis (LC) and hepatocellular carcinoma (HCC) [1]. The prevalence of HBV infections in Vietnam is high, ranging from 10 to 20% in the general population [2]. As a foreseeable result, the long-term consequences of HBV infection will constitute a huge burden for Vietnamese economic conditions and the health system in the next decades.

Interactions between host immune responses and HBV determine the clinical manifestations of the infection. Compelling evidence indicates that vitamin D signaling and VDR variants have a significant implication on susceptibility to HBV infection and subsequent liver diseases. In addition to its function in regulating calcium and bone homeostasis, various non-skeletal effects of vitamin D have been identified, including anti-inflammatory, anti-fibrotic and anti-tumor properties. The effects of vitamin D deficiency on various health conditions such as autoimmune diseases, malignancies and a plethora of infectious diseases have extensively been discussed [3–6]. Among communicable diseases, vitamin D deficiency is frequently observed in patients with hepatitis B and C (HCV)-related liver diseases and linked to unfavorable clinical outcomes as well as poor response to antiviral treatment in chronic hepatitis B [7–10] and chronic hepatitis C [11–13].

The active form of vitamin D (calcitriol, 1,25(OH)2D) can modulate both innate and adaptive immune responses through binding to the vitamin D receptor (VDR) [14–17]. The VDR is a member of the nuclear receptor family of transcription factors [18], which is particularly expressed on macrophages, T cells, and B cells [14, 15, 19], but also in more than 30 other types of tissues [20]. It has been suggested that Vitamin D-VDR signaling regulates the expression of more than 900 genes involved in a wide array of physiological functions [17]. Therefore, VDR is considered a powerful modulator of pathophysiological mechanisms in several human diseases such as cancers, metabolic disorders and infectious diseases, including viral hepatitis [6, 11, 21–25].

The *VDR* gene consists of eight exons (exon 2–9) encoding the VDR structural component and six alternatively spliced untranslated exons (1a-1f) [26]. Among numerous single nucleotide polymorphisms (SNPs) identified in the *VDR* gene, the four common variants FokI G/A (rs2228570, exon 2), BsmI C/T (rs1544410, intron 8), ApaI A/C (rs7975232, intron 8) and TaqI T/C (rs731236, exon 9) have been examined in many studies in different ethnic groups in order to identify associations of these variants with HBV infection and liver disease outcomes [27–34]. However, the results were inconsistent. Our study in Vietnamese patients chronically infected with HBV aimed to investigate the association of these *VDR* polymorphisms with susceptibility to HBV infection and liver disease outcomes.

## Methods

### Patient population

Four hundred forty-three Vietnamese HBV-infected patients were enrolled for this case-control study at the 108 Military Central Hospital, Hanoi, Vietnam, between 2012 and 2014. HBV patients were assigned to the different clinical subgroups based on clinical manifestations. Briefly, CHB patients (*n* = 183) were characterized based upon clinical symptoms such as fatigue, anorexia, jaundice, hepatomegaly, hard density of the liver, splenomegaly, hyperbilirubinemia, elevated levels of AST and ALT and HBsAg positivity for more than 6 months. HBV-infected LC patients (*n* = 89) presented clinical manifestations such as anorexia, nausea, vomiting, malaise, weight loss, abdominal distress, jaundice, edema, cutaneous arterial spider angiomas, ascites, shrunken liver, splenomegaly, hyperbilirubinemia, elevated levels of AST and ALT, prolonged serum prothrombin time, and decreased serum albumin. HCC patients (*n* = 171) were diagnosed based on the combination of clinical manifestations, imaging modalities (abdominal ultrasound, MRI, CT scanner), and histological diagnoses. None of the patients had a history of alcohol or drug abuse. In addition, 238 healthy HBsAg-negative individuals were randomly enrolled from the hospital’s blood bank as controls (HC group). They were students in the universities or employers in manufacturing companies located within or nearby Hanoi. None of the HCs had a history of a liver disease, nor were they immunosuppressed. All participants were negative for anti-HCV and anti-HIV antibodies by using commercial ELISA assays (Diagnostic automation/Cortez Diagnostics, Inc., Woodland Hills, California, USA). Patients were assigned to three subgroups based on their clinical manifestations and laboratory evaluations as previously described [7] including CHB (*n* = 183), LC (*n* = 89), and HCC patients (*n* = 171). All patients and controls came from Northern Vietnam and belonged to the Kinh ethnicity. Biomedical measurements (ALT and AST enzyme levels, alpha fetoprotein (AFP), platelet count (PLT), total and direct bilirubin, albumin, prothrombin, and HBV DNA loads) were collected from medical records for each patient. Total vitamin D levels were also measured in serum samples from patients and controls using a commercial ELISA kit (Gentaur, Kampenhout, Belgium) according to the manufacturer’s instructions. In addition, Peripheral blood was collected from all study subjects, and plasma was immediately separated. Samples were stored at − 80 °C until further use.

### Genotyping of VDR polymorphisms by sequencing and amplification-refractory mutation system (ARMS-PCR*)*

The *VDR* variants TaqI, FokI, ApaI*,* BsmI were genotyped by direct sequencing and validated by amplification-refractory mutation system (ARMS) methods. First, a set of 244 samples (194 HBV patients, 50 HCs) were genotyped for 4 SNPs. Subsequently, 96 samples out of these 244 samples that had all genotypes of each SNP were chosen for validation by ARMS-PCR. The genotyping results of the 96 samples generated through the ARMS PCR assay were completely matched against the results of direct sequencing. After validation, the remaining samples of 249 HBV patients and 199 HCs were genotyped by applying our validated ARMS PCR assay, which is a low-cost genotyping technique and suitable for common laboratory settings in Vietnam.

#### Sanger sequencing approach

Genomic DNA was isolated from whole blood using a DNA purification kit (Qiagen, Hilden, Germany). Four different fragments, consisting of recognized VDR variants were amplified by PCR. The primer sequences are given in Table 1. PCR amplification was carried out in a 25 μl volume containing 1X PCR buffer, 0.2 mM dNTPs, 1 mM MgCl_2_, 0.15 mM of each primer, 1 unit of Taq polymerase and 50 ng of genomic DNA. Cycling conditions were denaturation at 95 °C for 5 min, followed by 40 cycles of three-step cycling with denaturation (94 °C, 30 s), annealing for 35 s (annealing temperature for each primer pair given in Table 1), extension (72 °C, 45 s) and a final extension step (72 °C, 7 min). PCR products were purified by Exo-SAP-IT (USB, Affymetrix, USA) and 5 μl of products were used as sequencing templates (BigDye terminator v.1.1 cycle sequencing kit, ABI 3130XL DNA sequencer; Applied Biosystems, Foster City, USA). Determination of genotype was performed using the Geneious software (https://www.geneious.com/).

**Table 1:** Primers used for ARMS assays and DNA sequencing to genotype four VDR polymorphisms

SNPs	Sequences	Genotype-based product size
TaqI (rs731236)		
Outer^$^	F: 5′ GCT GCC GTT GAG TGT CTG TGT GGG TG 3’	
	R: 5′ ACA AGG GGC GTT AGC TTC ATG CTG CAC TC 3’	TT: 415 and 300 bp
Inner	T/F: 5′ CAG GAC GCC GCG CTG CTT 3’	TC: 415, 300 and 150 bp
	C/R: 5′ CGG TCC TGG ATG GCC GCG 3’	CC: 415 and 150 bp
BsmI (rs1544410)		
Outer^$^	F: 5′ GTG GTG TGT GGA CGC TGA GGT G 3’	
	R: 5′ TTC CTT GAG CCT CCA GTC CAG GAA AG 3’	TT: 458 and 350 bp
Inner	T/F: 5′ GGG CCA CAG ACA GGC CTA CA 3’	TC: 458, 350 and 150 bp
	C/R 5′ CAG AGC CTG AGT ATT GGG AAC GC 3’	CC: 458 and 150 bp
ApaI (rs7975232)		
Outer^$^	F: 5′ ATG GAA GGA CCT AGG TCT GGA TCC TAA ATG C 3’	
	R: 5′ GCT GCA CTC AGG CTG GAA GGA G 3’	TT: 420 and 280 bp
Inner	T/F: 5′ GTG GTG GGA TTG AGC AGT GAA GT 3’	GT: 420, 280 and 180 bp
	G/R: 5′ ACA GGA GCT CTC AGC TGG ACC 3’	GG: 420 and 180 bp
FokI (rs2228570)		
Outer^$^	F: 5′ ATG CCC ACC CTT GCT GAG CTC 3’	
	R: 5′ ATC TGG AGC TGA GAG GAG GGA AAA GAA GA 3’	GG: 477 and 360 bp
Inner	G/F: 5′ GCC TGC TTG CTG TTC TTA CAG GAA C 3’	GA: 477, 360 and 160 bp
	A/R: 5′ CTG GCC GCC ATT GCC TTC A 3’	AA: 477 and 160 bp

**(**^**$**^**)** Outer primers were used for direct sequencing to genotype VDR polymorphisms

#### Amplification refractory mutation system (ARMS) PCR assay

**Fig. 1:**
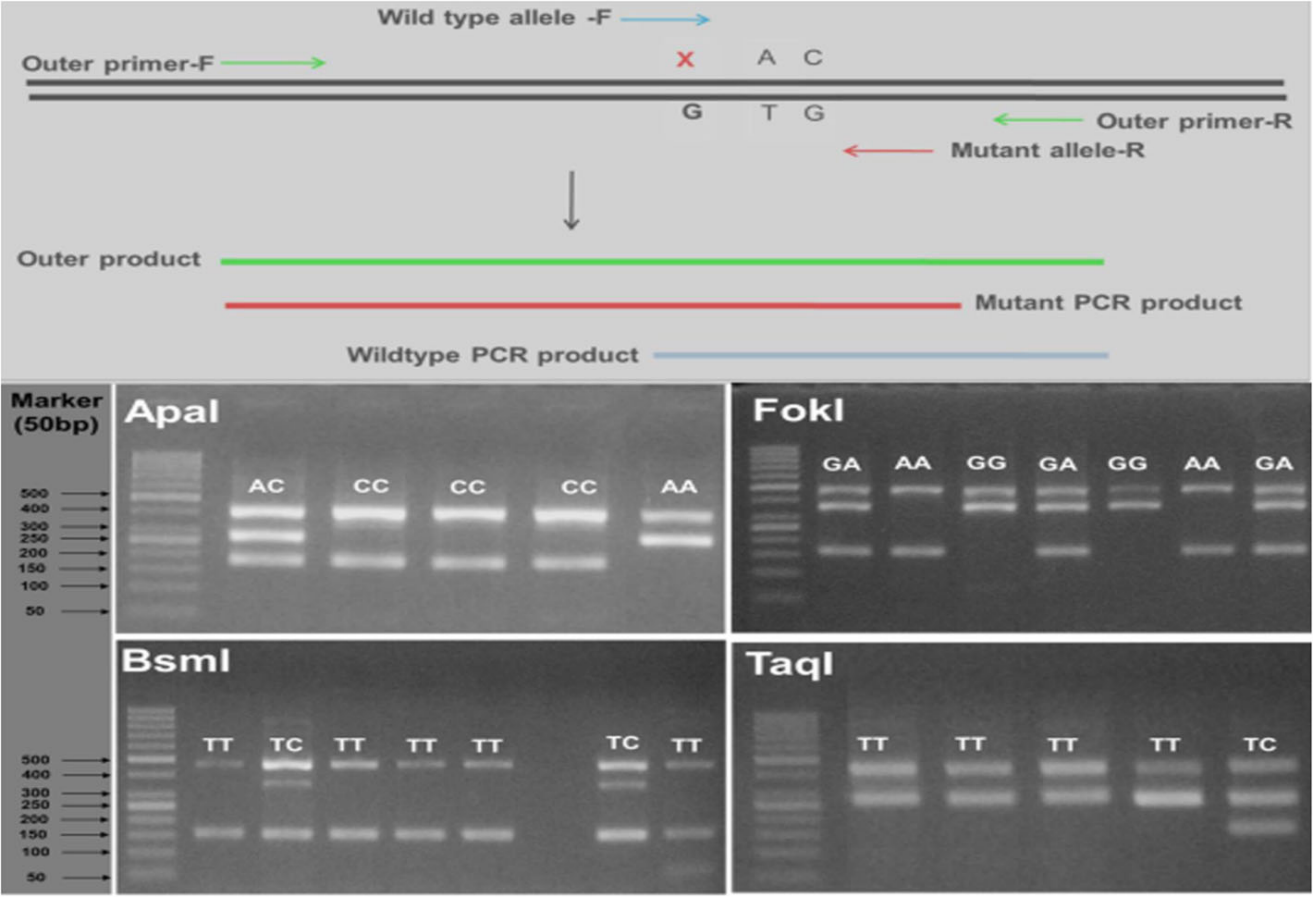
Illustration of ARMS assay for analyzing VDR polymorphisms. Upper panel: Schematic diagram of the principle of ARMS to detect mutant and wildtype alleles using allele specific primers. Lower panel: Gel electrophoresis analyses to distinguish genotypes of each VDR polymorphism. (**i**) ApaI polymorphism: AA genotype: 2 bands, 420 and 280 bp; CC genotype: 2 bands, 420 and 180 bp; AC genotype: 3 bands, 420, 280 and 180 bp. (**ii**) FokI polymorphism: GG genotype: 2 bands, 477 and 360 bp; AA genotype: 2 bands, 477 and 160 bp; GA genotype: 3 bands, 477, 360 and 160 bp. (**iii**) BsmI polymorphism: TT genotype: 2 bands, 458 and 150 bp; TC genotype: 3 bands, 458, 350 and 150 bp. (**iv**) TaqI polymorphism: TT genotype: 2 bands, 415 and 300 bp; TC genotype: 3 bands, 415, 300 and 150 bp

**Fig. 2:**
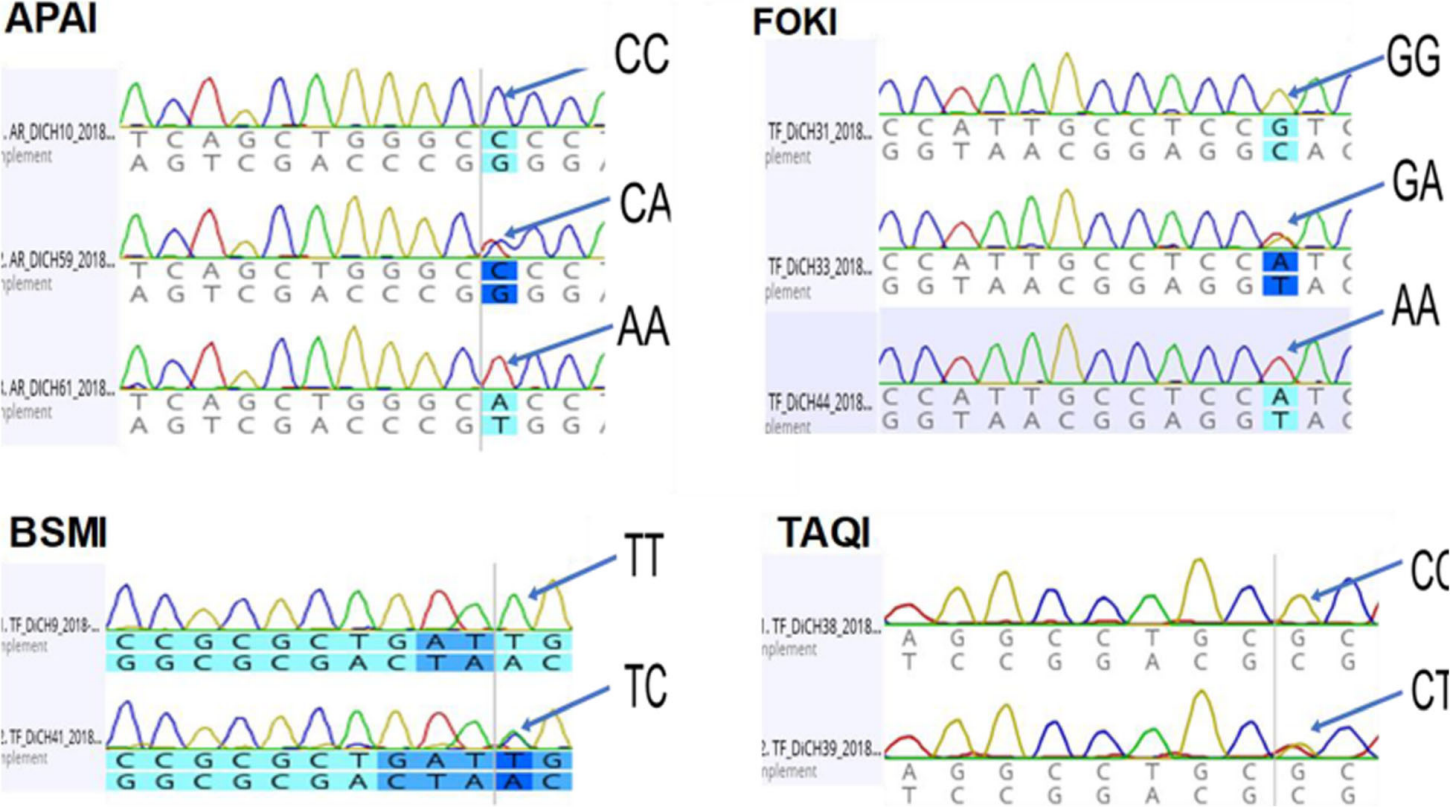
Illustration of sequencing results of the four VDR polymorphisms. (**1**) ApaI polymorphism, arrows indicate genotypes CC, CA and AA. (**2**) FokI polymorphism, arrows show genotypes GG, GA and AA. (**3**) BsmI polymorphism, arrows show genotype CC and CT. (**4**) TaqI polymorphism, arrows show genotypes TT and TC

ARMS is a PCR application in which DNA is amplified by allele specific primers. Heterozygosity or homozygosity is differentiated by using ARMS primers (inner primers) for the mutant/polymorphic and the normal (wild type) alleles. The primer sequences and the principles of ARMS assay are listed in Table 1 and Fig. 1, respectively. After the PCR assay, the mixtures of products were resolved by 1% agarose gel electrophoresis. This procedure reliably allowed to determine individual genotypes based on the sizes of amplicons (Fig. 1) and by sanger sequencing (Fig. 2).

### Statistical and genetic analysis

Statistical analyses were performed using R version 3.1.2 (http://www.r-project.org). Genotype and allele frequencies were determined by simple gene counting. Deviations from Hardy-Weinberg equilibrium were calculated for each group. Chi-square, Kruskal-Wallis and Mann-Whitney-Wilcoxon tests were used to compare differences between groups for qualitative or quantitative variables where appropriate. For genetic analyses, we used univariate analyses, and a multivariate logistic regression model adjusted for age and gender to test for associations between *VDR* variants and HBV-related liver diseases, applying different genetic models (codominant, dominant, recessive and overdominant models). The level of significance was set at *P* < 0.05.

## Results

### Baseline characteristics of patients and controls

**Fig. 3:**
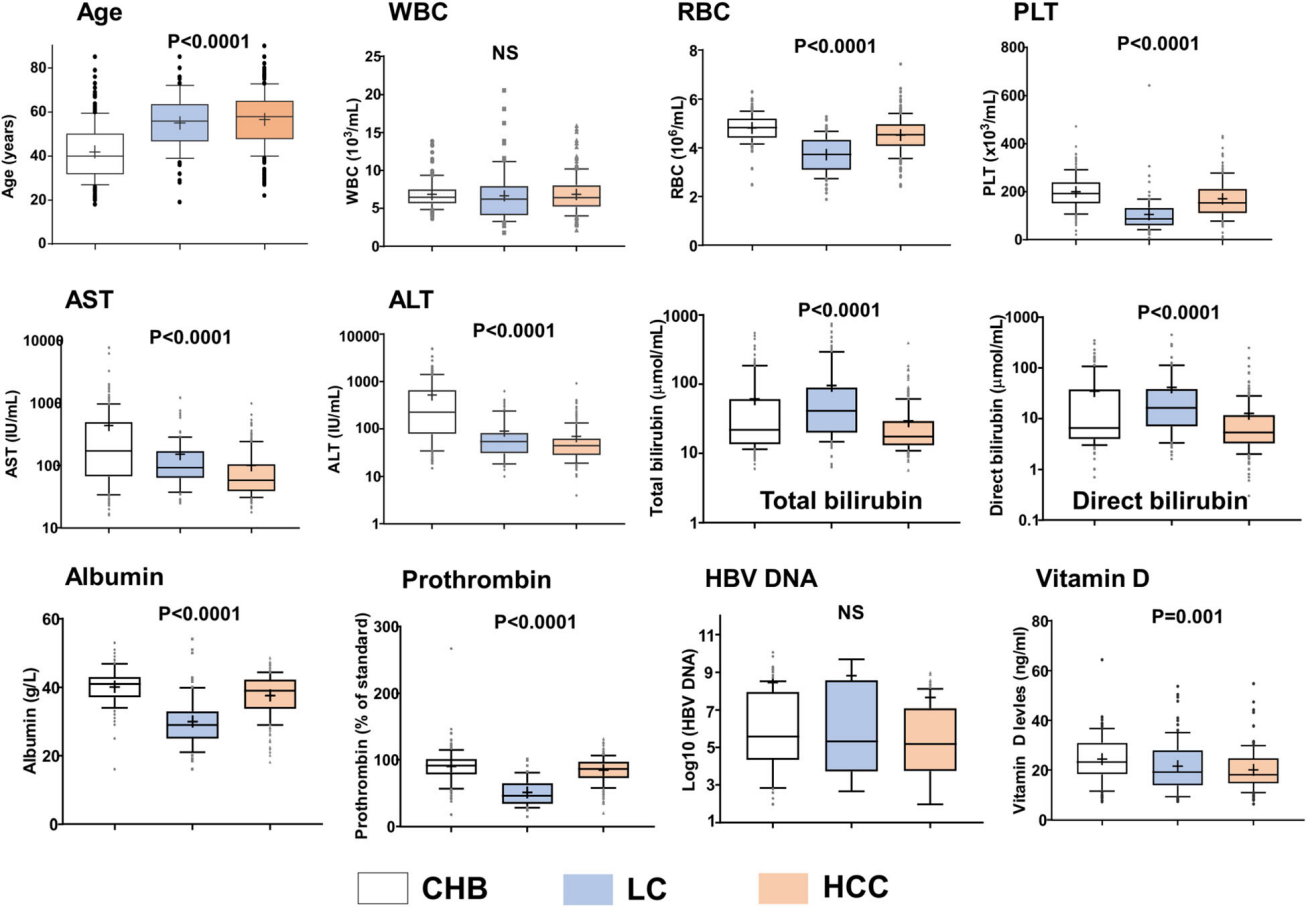
Comparison of clinical parameters in patient subgroups. CHB, chronic hepatitis B; LC, liver cirrhosis; HCC, hepatocellular carcinoma; PLT, platelets. AST and ALT, aspartate and alanine amino transferase; WBC, white blood cells; RBC, red blood cells; PLT, platelet; IU, international unit; NS, not significant. Box-plots illustrate medians with 25 and 75 percentiles with whiskers to 10 and 90 percentiles. *P* values were calculated by Kruskal-Wallis test

The baseline characteristics of the 473 HBV-infected patients and 238 HCs are given in Table 2 and Fig. 3. The majority of patients and HCs were males (89 and 67%, respectively). HCs were younger than patients. Liver enzyme levels (ALT and AST) were higher in patients with CHB compared to those with LC and HCC (*P* < 0.0001). Patients with LC had significantly lower levels of red blood cell, platelet, albumin and prothrombin but higher levels of bilirubin compared to those without LC (*P* < 0.0001). AFP levels were significantly higher among the HCC group compared to the CHB and LC groups (*P* < 0.0001). There was no significant difference in HBV DNA levels between patient subgroups.

**Table 2:** Clinical profiles of HBV-infected patients and healthy controls

Clinical characteristics	Patients (*n* = 473)	Controls (*n* = 238)
Age (years)	51 (18–90)	30 (18–55)
Male/Female	420/53	159/79
HBsAg	Positive	Negative
Anti-HCV	Negative	Negative
Anti-HIV	Negative	Negative
AFP (IU/mL)	11.78 (0.8–400)	< 5
HBV DNA (copies/mL)	269,408 (100–11,300,000,000)	NA
WBC (×10^3^/mL)	6.4 (1.8–20.5)	NR
RBC (× 10^6^/mL)	4.1 (1.9–7.43)	NR
PLT (×10^3^/mL)	157 (6.7–641)	NR
AST (IU/mL)	90 (16–7700)	NR
ALT (IU/mL)	61.25 (4–4908)	NR
Total Bilirubin (umol/mL)	20.7 (5.7–733)	NR
Direct Bilirubin (umol/mL)	6.7 (1–450)	NR
Albumin (g/L)	38.7 (16–54)	ND
Prothrombin (% of standard)	85 (14–267)	ND
Vitamin D levels (ng/ml)	20.42 (6.4–64.4)	20 (11.6–85.6)

*PLT* platelets, *AST* and *ALT* aspartate and alanine amino transferase, *WBC* white blood cells, *RBC* red blood cells, *IU* international unit, *NR* normal range, *NA* not applicable, *ND* not done. Values given are medians and ranges

Vitamin D levels were measured randomly in 50 healthy individuals and in 286 HBV patients (CHB = 92, LC = 89, HCC = 105). The levels of vitamin D were lower in HBV patients compared to healthy individuals, but the difference did not reach statistical significance (*P* > 0.05). However, in the patient group, vitamin D levels were significantly decreased according to progression of liver disease as found higher in CHB patients, followed by the LC and HCC groups (Fig. 3).

### VDR genotyping and the association of VDR polymorphisms with HBV infection and the progression of liver diseases

The genotype and allele frequencies of four SNPs in the *VDR* gene of the 473 HBV-infected patients and 238 HCs and the comparisons between the different patient groups are shown in Table 3. The genotype frequencies of the SNPs among HBV patients and HCs were in Hardy-Weinberg equilibrium (*P* > 0.05). In order to analyze the association of *VDR* SNPs with susceptibility to HBV infection, we compared the genotype frequencies between HBV patients and HCs. There were no differences in genotype frequencies between the two groups, indicating that TaqI, FokI, ApaI, BsmI are not associated with HBV infection per se*.*

**Table 3:** Distribution of genotype and allele frequencies of VDR variants in controls, cases and patient subgroups

VDR polymorphisms	Genotype frequency n, (%)						P*	Allele frequency n, (%)				P*	Comparisons
ApaI (rs7975232)	CC		AC		AA			C		A			
Controls	114	(48.7)	106	(45.3)	14	(6)	NS	334	(71.4)	134	(28.6)	NS	Cases vs. controls
Cases	203	(47.8)	183	(43.2)	38	(9)		589	(70)	259	(30)		
CHB	76	(42.2)	94	(52.2)	10	(5.6)	NS	246	(68.3)	114	(31.7)	NS	LC vs. CHB
LC	41	(50.6)	32	(39.5)	8	(9.9)	NS	114	(70.3)	48	(29.7)	NS	HCC vs. LC
HCC	86	(52.8)	57	(35)	20	(12.3)	0.0022	229	(70.2)	97	(29.8)	NS	HCC vs. CHB
non-HCC	117	(44.8)	126	(48.3)	18	(6.9)	0.013	360	(69)	162	(31)	NS	HCC vs. Non-HCC
FokI (rs2228570)	GG		GA		AA			G		A			
Controls	49	(23)	116	(54.5)	48	(22.5)	NS	214	(50.2)	212	(49.8)	NS	Cases vs. controls
Cases	108	(25)	233	(54)	91	(21)		449	(52)	415	(48)		
CHB	49	(27.8)	91	(51.7)	36	(20.5)	NS	189	(53.7)	163	(46.3)	NS	LC vs. CHB
LC	12	(15.4)	47	(60.3)	19	(24.3)	NS	71	(45.5)	85	(55.5)	NS	HCC vs. LC
HCC	47	(26.4)	95	(53.4)	36	(20.2)	NS	189	(53)	167	(47)	NS	HCC vs. CHB
non-HCC	61	(24)	138	(54.3)	55	(21.7)	NS	260	(51.2)	248	(48.8)	NS	HCC vs. Non-HCC
TaqI (rs731236)	TT		TC		CC			T		C			
Controls	177	(90.8)	18	(9.2)	0	(0)	NS	372	(95.4)	18	(4.6)	NS	Cases vs. controls
Cases	423	(91.4)	40	(8.6)	0	(0)		886	(95.7)	40	(4.3)		
CHB	168	(92.3)	14	(7.7)	0	(0)	NS	350	(96.2)	14	(3.8)	NS	LC vs. CHB
LC	81	(91.1)	8	(8.9)	0	(0)	NS	170	(95.5)	8	(4.5)	NS	HCC vs. LC
HCC	174	(90.6)	18	(9.4)	0	(0)	NS	366	(95.4)	18	(4.6)	NS	HCC vs. CHB
non-HCC	249	(91.9)	22	(8.1)	0	(0)	NS	520	(96)	22	(4)	NS	HCC vs. Non-HCC
BsmI (rs1544410)	CC		CT		TT			C		T			
Controls	196	(92.5)	16	(7.5)	0	(0)	NS	408	(96.2)	16	(3.8)	NS	Cases vs. controls
Cases	404	(94)	26	(6.0)	0	(0)		838	(97)	26	(3)		
CHB	162	(92.6)	13	(7.4)	0	(0)	NS	337	(96.3)	13	(3.7)	NS	LC vs. CHB
LC	71	(93.4)	5	(6.6)	0	(0)	NS	147	(96.7)	5	(3.3)	NS	HCC vs. LC
HCC	171	(95.6)	8	(4.4)	0	(0)	NS	350	(97.7)	8	(2.3)	NS	HCC vs. CHB
non-HCC	233	(92.8)	18	(7.2)	0	(0)	NS	484	(96.4)	18	(3.6)	NS	HCC vs. Non-HCC

*CHB* chronic hepatitis B, *LC* liver cirrhosis, *HCC* hepatocellular carcinoma; Cases = all HBV infected patients; non-HCC, CHB + LC, n = number of chromosomes; *OR* adjusted Odds Ratio, *NS* not significant, (*) Chi squared test

Chi-squared tests were applied to examine whether there were differences in genotype and allele frequencies of each *VDR* polymorphism in pairwise comparisons [cases vs. HCs; HCC vs. LC; LC vs. CHB; HCC vs. CHB and HCC vs. non-HCC (or CHB + LC)]. We found a significant difference in genotype frequencies of the ApaI polymorphism between the CHB and the HCC groups and between HCC and non-HCC groups (*P* = 0.002 and 0.013, respectively, Table 3). No associations were observed for other comparisons and for TaqI, BsmI and FokI polymorphisms.

Next, we applied logistic regression analyses for different genetic models to compare the genotype frequencies of the ApaI variant between the CHB and HCC groups. Genotype CA was less frequent in HCC, compared to CHB patients in codominant and overdominant models (codominant model, OR = 0.5, 95%CI = 0.3–0.84, adjusted *P* = 0.004; overdominant model, OR = 0.46, 95%CI = 0.27–0.76, adjusted *P* = 0.0023, Table 4). In the recessive model, the genotype AA was significantly more frequent in HCC compared to CHB patients (OR = 2.56, 95%CI = 1.01–6.48, *P* = 0.04). Conversely, in the dominant model, genotypes CA and AA were less frequent in HCC than in CHB patients (OR = 0.62, 95%CI = 0.37–0.94, *P* = 0.04) (Table 4). When genotype frequencies of the ApaI variant were compared between HCC and non-HCC groups, the genotype CA was less frequent in HCC compared to non-HCC patients in codominant model (*P* = 0.04, Table 4).

**Table 4:** Association of ApaI variant with liver disease progression

ApaI SNP	CHB		CHB + LC (non-HCC)		HCC		HCC vs. CHB		HCC vs. non-HCC	
	n	%	n	%	n	%	OR (95%CI)	P*	OR (95%CI)	P*
Codominant										
CC	76	42.2	117	44.8	86	52.8	1	Ref	1	Ref
CA	94	52.2	126	48.3	57	35	0.5 (0.3–0.84)	**0.004**	0.6 (0.4–0.98)	**0.04**
AA	10	5.6	18	6.9	20	12.3	1.84 (0.7–4.8)	0.206	1.4 (0.7–3.0)	0.189
Dominant										
CC	76	42.2	117	44.8	86	52.8	1	Ref	1	Ref
CA + AA	104	57.8	144	55.2	77	47.2	0.62 (0.37–0.94)	**0.04**	0.72 (0.47–1.1)	0.13
Recessive										
CC + CA	170	94.4	243	93.1	143	87.7	1	Ref	1	Ref
AA	10	5.6	18	6.9	20	12.3	2.56 (1.01–6.48)	**0.04**	1.74 (0.8–3.65)	0.14
Overdominant										
CC + AA	86	47.8	135	51.7	106	65	1	Ref	1	Ref
CA	94	52.2	126	48.3	57	35	0.46 (0.27–0.76)	**0.0023**	0.6 (0.38–0.90)	**0.017**

*CHB* Chronic hepatitis B, *LC* Liver cirrhosis, *HCC* Hepatocellular carcinoma; Non-HCC, CHB + LC; n = number of chromosomes; *OR* adjusted Odd Ratio; ORs and *P* values were calculated by using binary logistic regression model adjusted for age and gender. (*) Logistic regression model adjusted for age and gender. Bold values present the statistical significance

### Association of VDR variants with clinical parameters

**Fig. 4:**
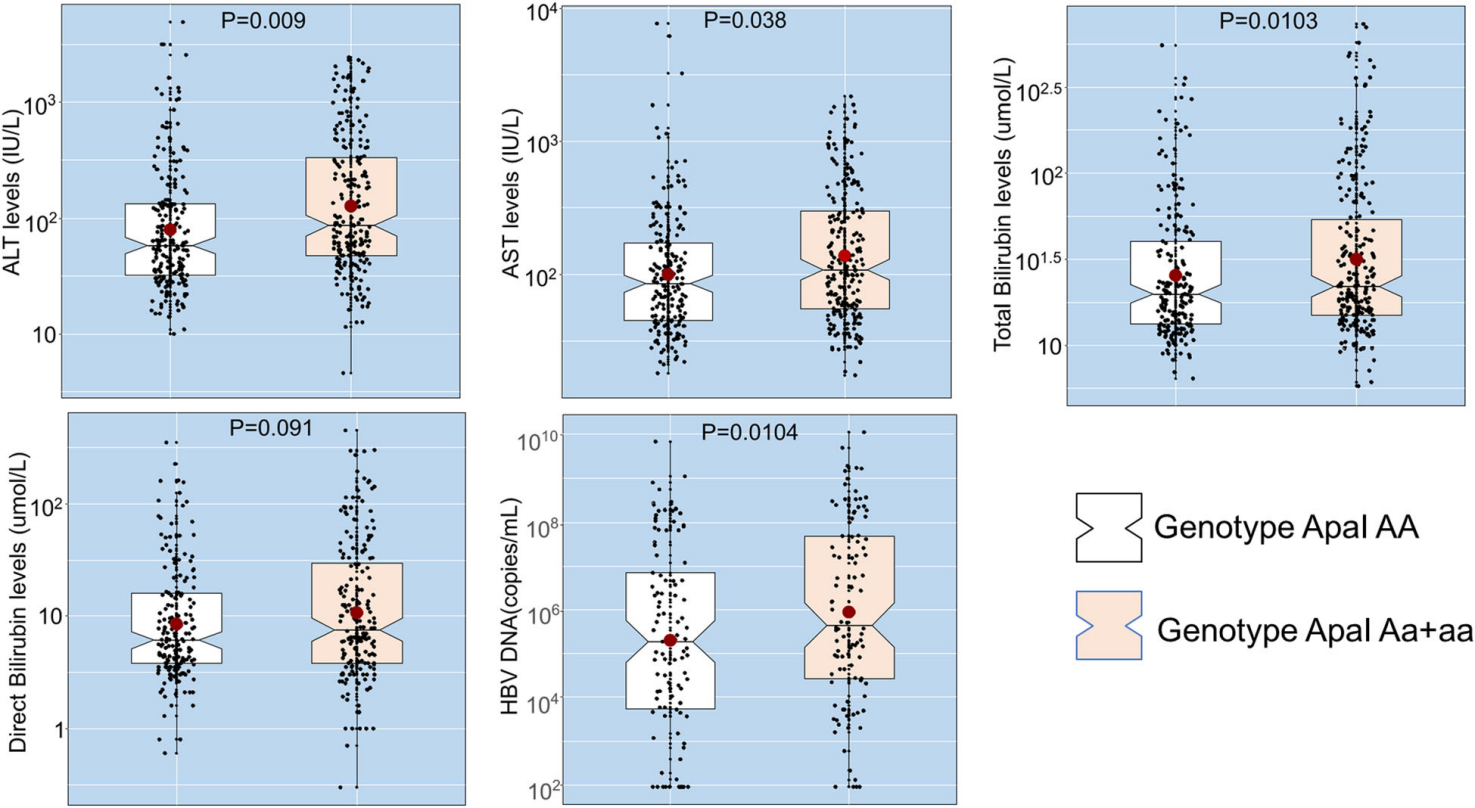
Association of ApaI polymorphism with clinical parameters in HBV patients. CHB, chronic hepatitis B; LC, liver cirrhosis; HCC, hepatocellular carcinoma; PLT, platelets; AST and ALT, aspartate and alanine amino transferase; WBC, white blood cells; RBC, red blood cells; PLT, platelets; IU, international unit; NS, not significant. Box-plots illustrate medians with 25 and 75 percentiles with whiskers to 10 and 90 percentiles. *P* values were calculated by Man-Whitney Wilcoxon test

In order to study the influence of *VDR* variants on the clinical outcome of HBV-related liver diseases we compared laboratory parameters among HBV-infected patients with different genotypes of each of the *VDR* variants. Pathological liver function as indicated by high levels of AST, ALT, total and direct bilirubin were observed rather in HBV patients carrying the genotypes ApaI CA or AA than in those with the genotype ApaI CC (*P* < 0.05) (Fig. 4). HBV DNA levels were significantly lower in patients with the genotype ApaI CC compared to those with either ApaI CA or AA (*P* = 0.0104) (Fig. 4). Platelet counts, albumin and prothrombin levels were lower in patients with *VDR* ApaI CC compared to those with *VDR* ApaI CA or AA. The difference was, however, not statistically significant (data not shown).

**Fig. 5:**
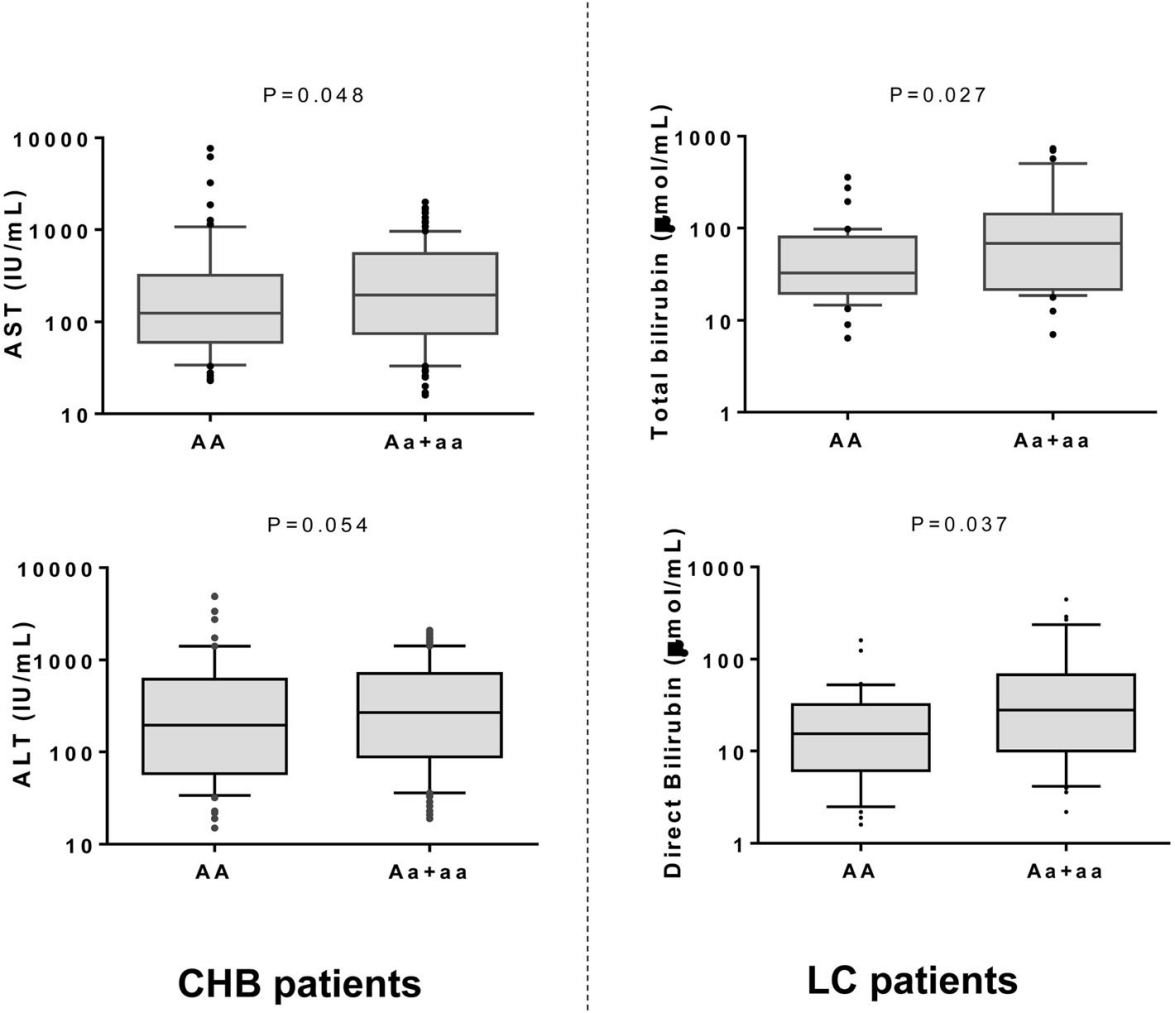
Distribution of liver enzymes and bilirubin levels in CHB and LC patients. CHB, chronic hepatitis B; LC, liver cirrhosis; AST and ALT, aspartate and alanine amino transferase; IU, international unit. Box-plots illustrate medians with 25 and 75 percentiles with whiskers to 10 and 90 percentiles. *P* values were calculated by Man-Whitney Wilcoxon test

When we compared the association of the ApaI variant with laboratory parameters in the subgroups of HBV patients, we found that AST and ALT levels were lower in individuals with the genotype CC than in those with genotypes CA and AA among the CHB patients (*P* = 0.048 and 0.054, respectively). Total bilirubin and direct bilirubin levels were significantly higher in LC patients with ApaI CA or AA compared to those with the genotype CC (*P* = 0.027 and 0.037, respectively) (Fig. 5). No differences were observed when comparing the occurrence of BsmI, TaqI, and FokI variants with any clinical parameter in HBV patients and in the subgroups (data not shown).

### Correlation between VDR variants with serum vitamin D levels

We analyzed the association of four *VDR* variants with the serum levels of vitamin D in all HBV patients as well as in each subgroup. We did not observe associations of any of the *VDR* variants with vitamin D levels (data not shown).

## Discussion

Vitamin D plays a crucial role in the metabolism of calcium and bone homeostasis, but has also multiple properties in the regulation of immune responses through engagement with the VDR, a member of the nuclear receptor family of transcription factors expressed on many immune cells and other tissues [14, 15, 19]. Vitamin D-VDR signaling is involved in pathophysiological mechanisms in several human diseases, including viral hepatitis [6, 11, 21–25]. We investigated the functional role of the four common *VDR* SNPs TaqI (rs731236) FokI (rs2228570), ApaI (rs7975232) and BsmI (rs1544410) in Vietnamese HBV-infected patients and observed a significant association between ApaI variability and clinical presentation and progression of HBV-related liver disease. The major and minor allele frequencies observed in this study were compared to available data in the 1000 genomes project. The allele frequencies were ApaI [rs7975232: Major allele C = 0.7424; Minor allele A = 0.2576]; FokI [rs2228570: Major allele G = 0.5859; Minor allele A = 0.4141]; Taq1 [rs731236: Major allele T = 0.9596; Minor allele C = 0.0404] and for BsmI [rs1544410 Major allele C = 0.9596; Minor allele T = 0.0404]. The observed minor allele frequencies from this study corroborated well with the available data. Kinh ethnicity is a major ethnic population (> 87% ~ 77 million) in Vietnam. Based on mtDNA data, all Vietnamese carry South East Asian haplotypes, which show a limited geographic and ethnic stratification [35], therefore no population substructure is foreseen.

Several studies have investigated the biological functions of the four *VDR* variants FokI (exon 2), BsmI (intron 8), ApaI (intron 8) and TaqI (exon 9) and their correlation with HBV infection [27–34]. However, the relationship between distinct *VDR* variants and HBV infection and liver disease progression has remained controversial. Regarding the association of these polymorphisms with the risk of HBV infection, a recent meta-analysis including 15 studies could not reveal any evidence of correlation between TaqI, ApaI and BsmI polymorphisms with HBV infection; correlations were only observed for the FokI polymorphism [25].

Five of the 15 studies in the above-mentioned meta-analysis have assessed the association of ApaI polymorphisms with HBV infection; however, no correlation was observed (CC vs. CA and AA). This finding corresponds to the results of our present study (Table 3). On the other hand, there is not much information available on the ApaI polymorphism and progression of HBV-related liver diseases. Two previous studies have shown that ApaI polymorphism did not show positive association with the risk and clinicopathological features of HCC [33, 36]. To our knowledge, our present study is the first to show an independent association between the *VDR* ApaI AA genotype and an increased risk of HBV-related development of HCC with a significant OR of 2.56 when comparing the HCC with the CHB group. An association of the ApaI CC genotype with the development of HCC among chronic hepatitis C patients has previously been demonstrated [37]. Allele ApaI C appears to contribute to a decreased susceptibility to HCV infection in a high-risk Chinese population [38]. A recent study has shown that the ApaI polymorphism was associated with the severity of liver cirrhosis caused by various etiologies [39].

Among numerous polymorphisms in the *VDR* gene, the FokI polymorphism is of great importance, as this missense variant causes a change of the start codon ATG to ACG, resulting in a modification of the VDR protein by three amino acids [40]. The modified protein, encoded by the FokI G allele, binds more efficiently to vitamin D than does the longer version coded by the A allele [26, 40]. It was suggested that the change in the VDR structure may alter the biological function of VDR-vitamin D transcriptional signaling and may contribute to carcinogenesis, autoimmunity and susceptibility to infectious diseases, including viral hepatitis [34, 41]. As shown in the above-mentioned meta-analysis, eight studies including a total of 2516 cases, 1088 controls were subjected to the evaluation of the role of the FokI polymorphism in HBV infection. The FokI polymorphism was significantly associated with HBV infection in a codominant model [25].

Three other studies have investigated possible association of the FokI polymorphism with liver cancer and have suggested that the FokI polymorphisms might be used as a molecular marker to predict the risk and to evaluate the disease severity of HCC in HBV-infected patients [33, 34, 36]. Triantos et al. reported that the occurrence of the GG genotype was also an independent prognostic marker of survival, providing a possible protective role in LC patients [39]. Other studies have been conducted to investigate the potential role of the FokI variant in response to treatment with pegylated interferon in chronic hepatitis B and chronic hepatitis C [42, 43]. These studies have shown that the FokI polymorphism was an independent factor predicting success of PEG-IFN treatment response in HBV- or HCV-infected patients. Although these studies demonstrated the potential role of FokI variability in HBV infection and progression of liver diseases as well as response to antiviral treatment, our study could not confirm any correlation between the FokI polymorphism with either susceptibility to HBV infection or progression of HBV related-liver diseases.

The TaqI polymorphism has widely been studied in several malignant diseases such as breast cancer, prostate cancer, skin cancer, colorectal cancer, liver cancer and other malignancies [41, 44]. However, any effects of this variant on the development of cancer remain inconclusive. For instance, two meta-analyses have assessed the association of the TaqI polymorphism with the development of colorectal cancer, yielding inconsistent results [45, 46]. While Serrano et al. reported that the genotype CC increases the risk of colorectal cancer by 43%, but not for other malignancies [45], Sheng et al. concluded that no association between this genotype and susceptibility to colorectal cancer exists [46]. With regard to the association of the TaqI polymorphism with liver cancer, our study clearly confirms other studies, showing no significant association between the TaqI polymorphism and liver disease progression as well as development of HCC in both HBV and HCV infections [33, 37, 47].

Data on BSMI polymorphisms in HBV infection are scanty. Only three studies on associations of the BSMI polymorphism with HBV infection have been published to date [33, 48, 49]. These studies were included in the meta-analysis [25] and showed that BsmI was not a reliable indicator for the risk of HBV infection. In line with these studies, we did not find any association between *VDR* polymorphisms and HBV infection, as well as progression of liver disease. Unlike the FokI variant, TaqI and BsmI variability does not influence the function of VDR expression [48]. This may explain why TaqI and BsmI polymorphisms were not associated with either HBV infection or disease progression in most studies conducted so far.

Vitamin D levels and liver disease progression and the response to antiviral therapy in HBV and HCV patients have previously been described [49]. Our previous study has shown that vitamin D levels decreased according to the progression of HBV-related liver disease [7]. One limitation of the study is that Vitamin D levels was measured only in fewer healthy controls compared to a large group of HBV patients. In the present study, we did not find any association of VDR polymorphisms with vitamin D levels in HBV-infected patients. Nevertheless, the correlation between VDR polymorphisms and vitamin D serum levels is not yet well established or this association is weak only. The influences of VDR polymorphisms on the course as well as the pathogenesis of HBV-related liver diseases remain still controversial and need to be explored further.

## Conclusions

In this study, we examined the association of VDR polymorphisms TaqI, FokI, ApaI, and BsmI with HBV infection risk and with the progression of HBV-related liver diseases. Although there was no association between VDR polymorphisms with HBV infection risk, the ApaI polymorphism might be a genetic factor associated with the clinical outcome and disease progression in Vietnamese HBV infected patients.

## Data Availability

All data generated or analysed during this study are included in this published article. Additional data are however available from the authors upon reasonable request.

## Abbreviations

AFP: Alpha-fetoprotein
ARMS: Amplification-refractory mutation system
AST and ALT: Aspartate and alanine amino transferase
CHB: Chronic hepatitis B
HBsAg: Hepatitis B surface antigen
HBV: Hepatitis B virus
HC: Healthy control
HCC: Hepatocellular carcinoma
LC: Liver cirrhosis
PLT: Platelets
SNP: Single nucleotide polymorphism
VDR: Vitamin D receptor
